# Sex disparities and the risk of urolithiasis: a large cross-sectional study

**DOI:** 10.1080/07853890.2022.2085882

**Published:** 2022-06-08

**Authors:** Jin-Zhou Xu, Cong Li, Qi-Dong Xia, Jun-Lin Lu, Zheng-Ce Wan, Liu Hu, Yong-Man Lv, Xiao-Mei Lei, Wei Guan, Yang Xun, Shao-Gang Wang

**Affiliations:** a Department of Urology, Tongji Hospital, Tongji Medical College, Huazhong University of Science and Technology, Wuhan, China; b Department of Urology, Sun Yat-sen Memorial Hospital, Sun Yat-sen University, Guangzhou, China; c Health Management Center, Tongji Hospital, Tongji Medical College, Huazhong University of Science and Technology, Wuhan, China

**Keywords:** Urolithiasis, sex difference, age, China, cross-sectional study

## Abstract

**Background:**

Urolithiasis is one of the most common diseases in urology, with a lifetime prevalence of 14% and is more prevalent in males compared to females. We designed to explore sex disparities in the Chinese population to provide evidence for prevention measures and mechanisms of stone formation.

**Materials and methods:**

A total of 98232 Chinese individuals who had undergone a comprehensive examination in 2017 were included. Fully adjusted odds ratios for kidney stones were measured using restricted cubic splines. Multiple imputations was applied for missing values. Propensity score matching was utilised for sensitivity analysis.

**Results:**

Among the 98232 included participants, 42762 participants (43.53%) were females and 55470 participants (56.47%) were males. Patients’ factors might cast an influence on the development of kidney stone disease distinctly between the two genders. A risk factor for one gender might have no effect on the other gender. The risk for urolithiasis in females continuously rises as ageing, while for males the risk presents a trend to ascend until the age of around 53 and then descend.

**Conclusions:**

Patients’ factors might influence the development of kidney stones distinctly between the two genders. As age grew, the risk to develop kidney stones in females continuously ascended, while the risk in males presented a trend to ascend and then descend, which was presumably related to the weakening of the androgen signals.

**Key messages**
- We found that patients’ factors might cast an influence on the development of kidney stone disease distinctly between the two sexes.
- The association between age and urolithiasis presents distinct trends in the two sexes
- The results will provide evidence to explore the mechanisms underlying such differences can cast light on potential therapeutic targets and promote the development of tailored therapy strategies in prospect.

## Introduction

Urolithiasis is one of the most common diseases in urology, with a lifetime prevalence of 14%. Approximately 50% of stone formers will experience another stone episode within 5 years, regardless of stone composition [1]. Stone formers suffer from pain, financial burden and are at higher risk of hydronephrosis, chronic kidney disease, and renal cell carcinoma [2,3]. To relieve the suffering of patients, there is an imperious need to elaborate on factors affecting the stone formation and effective prevention measures.

Urolithiasis is more prevalent in males compared to females with a prevalence of 9% in females and 19% in males, although the difference is reported to be narrowing [4]. The development of kidney calculi presents obvious sex differences. In addition, both risk factors and consequences of urolithiasis are distinct in different genders [5]. Seolhye et al. manifested that non-alcoholic fatty liver disease is a risk factor for males but not for females [6]. Diabetes mellitus and obesity were also reported to have a stronger association with the risk for urolithiasis in males [7]. Although quite a few studies were devoted to unraveling the relationship between sex and the development of urolithiasis, limitations such as uncontrolled bias, and limited generalisation scope urge more research to facilitate the comprehension of gender differences in urolithiasis.

Therefore, we herein provide analyses to explore sex disparities in the Chinese population. Clarifying male-female differences and further exploring the mechanisms underlying such differences can cast light on potential therapeutic targets and promote the development of tailored therapy strategies in prospect.

## Patients and methods

### Study population

This study was part of the project Influencing Factors for Common Chronic Diseases among Chinese Population (IFCCDCP). Individuals who underwent a comprehensive test at the Health Management Centre of Tongji Hospital in 2017 were included. This study was approved by the institutional review board of Tongji Hospital, Tongji Medical College, and Huazhong University of Science and Technology (Approval ID: TJ-C20160115). The study conformed to the ethical guidelines of the Declaration of Helsinki. Informed consent was obtained from each participant.

**Figure 1.**
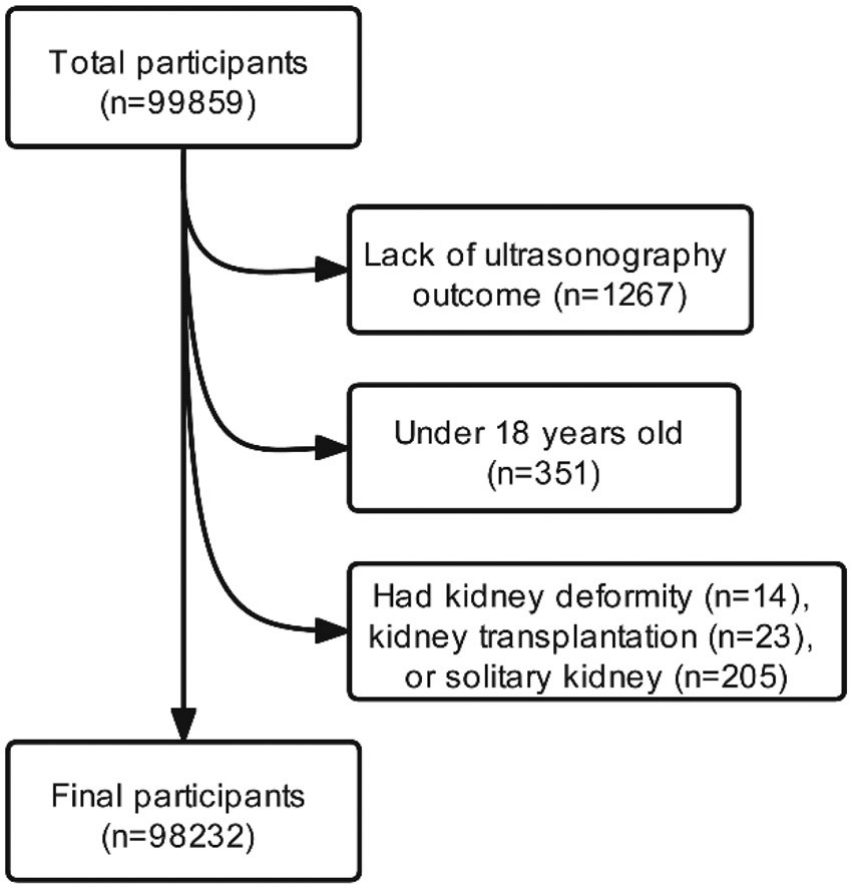
Flow of study participants in the study. After excluding 1627 participants who were lack of ultrasonography outcome (*n* = 1267), under 18 years old (*n* = 351), or had kidney deformity (*n* = 14), kidney transplantation (*n* = 23), solitary kidney (*n* = 205), those who completed a health examination were recruited (*n* = 98232).

After excluding 1627 participants who were lack of ultrasonography outcome (*n* = 1267), were under 18 years old (*n* = 351), or had kidney deformity (*n* = 14), kidney transplantation (*n* = 23), solitary kidney (*n* = 205), those who completed a health examination were recruited (*n* = 98232) (Figure 1).

### Measurement

Urolithiasis was diagnosed based on the ultrasonography outcome. Demographic characteristics and comorbidities including hypertension (HBP), diabetes (DM), fatty liver (FL), and coronary heart disease (CHD) were collected based on medical history. Presenting characteristics including body mass index (BMI), systolic blood pressure (SBP), and diastolic blood pressure (DBP) were measured when undergoing examination. Laboratory indices include alanine aminotransferase (ALT), aspartate aminotransferase (AST), total protein (TP), albumin (Alb), globulin (Glo), γ-glutamyl transpeptidase (GGT), serum creatinine (SCr), total bilirubin (TBIL), indirect bilirubin (IBIL), direct bilirubin (DBIL), total cholesterol (TC), high-density lipoprotein cholesterol (HDL), low-density lipoprotein cholesterol (LDL), triglycerides (TG), fasting blood glucose (Glu), and uric acid (UA), was tested from blood specimens. Urine pH (UpH) was acquired from a urinalysis, which can indicate the crystal type of kidney stones [8]. We used the CKD-EPI China equation with an adjusted coefficient of 1.1 for the Chinese population to calculate eGFR:

$$\mathrm{eGFR}=141\times {\min\left(\frac{\mathrm{SCr}}{\kappa },1\right)}^{\alpha }\times {\max\left(\frac{\mathrm{SCr}}{\kappa },1\right)}^{-1.209}\times 0.993^{\mathrm{Age}}\times 1.018(if\;\mathrm{female})\times 1.1$$

SCr is serum creatinine, *κ* is 0.7 for females and 0.9 for males, *α* is −0.329 for females and −0.411 for males, min is the minimum of SCr/κ or 1, and max indicates the maximum of SCr/κ or 1 [9].

### Statistical analyses

Multiple imputations were applied to evaluate the missing values. Data were presented as mean ± standard deviation or number (percentage). Logistic regression models were used to assess the association between selected variables and the occurrence of urolithiasis, presented by odds ratio (OR) (95% confidence interval [CI]). Models were sequentially adjusted for age and comorbidities including obesity (BMI <23.9, ≥23.9 kg/m^2^), HBP (present/absent), DM (present/absent), FL (present/absent), and CHD (present/absent) (model 1), plus and laboratory indices including HDL, LDL, TG, UA, Glu, UpH and eGFR (model 2).

Restricted cubic splines with 5 knots (5th, 27.5th, 50th, 72.5th, and 95th percentiles of age distribution) were applied to determine the dose-response relationship between age and urolithiasis presence. This relationship was also evaluated in both genders.

In age-stratified analysis (18–29, 30–44, 45–59, 60–74, ≥75 y, according to the WHO age segment method), the morbidity of urolithiasis in males and females was calculated. The OR for urolithiasis in each stratified age was quantified by logistic regression setting the group aged 18–29 y as reference.

For subgroup analysis, we applied logistic regression (model 2) to assess the OR of urolithiasis in males to females. Variables were grouped as follows: age (18–29, 30–44, 45–59, 60–74, ≥75 y), obesity (BMI <23.9, ≥23.9 kg/m^2^), HBP(present, absent), DM (present, absent), CHD (present, absent), FL(present, absent), SBP (<90, 90–120, ≥120 mmHg), DBP (<60, 60–90, ≥90 mmHg), eGFR (<90, 90–119, ≥120 mL/min/1.73m^2^), HDL (<1.0, ≥1.0 mmol/L), LDL (<3.4, ≥3.4 mmol/L), TG (<1.7, ≥1.7 mmol/L), UA (For females <4.88 and for males <5.55; For females ≥4.88 and for males ≥5.55, according to our former research) [10], Glu (<6.1, ≥6.1 mmol/L), UpH (<6.0, ≥6.0). The tests for interaction across subgroups were performed using the Wald test.

A sensitivity analysis was conducted applying propensity score matching. After matching, we repeated some of the former analyses and compared the results with those before matching. Considering that the sensitivity of ultrasonography for kidney stones is reduced for stones <3 mm, we excluded the samples whose stones were reported to be <3mm (*n* = 351), and performed logistic regression to minimise the influence of the low sensitivity of ultrasound on the reliability of our results [11]. R software (version 4.0.3) was used to perform all the statistical analyses. All *P*-values were two-tailed and *p* < .05 was considered statistically significant.

## Results

No significant difference exists before and after the multiple imputations (sTable 1). Table 1 shows the baseline characteristics of participants according to sex disparities. Among the 98232 included participants, 42762 participants (43.53%) were females and 55470 participants (56.47%) were males. The prevalence of urolithiasis was 11.4%, and the mean age was 41.22 ± 12.95 years at baseline. We also compared the information of participants concerning comorbidities, presenting characteristics, and laboratory indices (Table 1).

**Table 1.** Basic characteristics of included female and male participants.

Variables	All participants	Female	Male
	(*n* = 98232)	(*n* = 42762)	(*n* = 55470)
Urolithiasis^a^ present (%)	11211 (11.4)	3076 (7.2)	8135 (14.7)
Age, y	41.22 ± 12.95	40.43 ± 13.01	41.82 ± 12.86
Comorbidities			
Obesity present (%)	43025 (43.8)	11284 (26.4)	31741 (57.2)
Hypertension present (%)	8090 (8.2)	2671 (6.2)	5419 (9.8)
Diabetes present (%)	2349 (2.4)	682 (1.6)	1667 (3.0)
Coronary heart disease present (%)	529 (0.5)	170 (0.4)	359 (0.6)
Fatty liver present (%)	26217 (26.7)	5812 (13.6)	20405 (36.8)
Presenting characteristics			
BMI^b^, kg/m^2^	23.55 ± 3.38	22.25 ± 3.10	24.56 ± 3.24
SBP, mmHg	123.83 ± 17.94	119.16 ± 17.69	127.44 ± 17.29
DBP, mmHg	75.79 ± 12.03	71.96 ± 11.08	78.74 ± 11.90
Laboratory indices			
ALT, U/L	23.32 ± 22.20	16.71 ± 15.29	28.41 ± 25.17
AST, U/L	21.95 ± 12.46	19.82 ± 9.82	23.60 ± 13.95
TP, g/L	76.02 ± 3.96	76.21 ± 3.97	75.89 ± 3.95
Alb, g/L	46.11 ± 2.60	45.56 ± 2.48	46.53 ± 2.60
Glo, g/L	29.91 ± 3.57	30.64 ± 3.48	29.35 ± 3.54
GGT, U/L	31.05 ± 34.72	19.96 ± 17.38	39.60 ± 41.63
Scr, μmol/L	73.86 ± 18.48	60.21 ± 10.04	84.38 ± 16.53
eGFR^c^, mL/min/1.73m^2^	112.02 ± 17.23	117.63 ± 16.65	107.70 ± 16.40
TBIL, μmol/L	13.63 ± 5.47	12.34 ± 4.67	14.63 ± 5.82
IBIL, μmol/L	9.96 ± 4.11	9.07 ± 3.48	10.64 ± 4.41
DBIL, μmol/L	3.67 ± 1.70	3.27 ± 1.38	3.99 ± 1.85
TC, mmol/L	4.53 ± 0.87	4.49 ± 0.86	4.56 ± 0.88
HDL, mmol/L	1.28 ± 0.31	1.44 ± 0.30	1.16 ± 0.25
LDL, mmol/L	2.73 ± 0.75	2.63 ± 0.73	2.80 ± 0.75
TG, mmol/L	1.47 ± 1.28	1.12 ± 0.83	1.73 ± 1.49
UA, mg/dL	5.75 ± 1.61	4.64 ± 1.07	6.61 ± 1.42
Glu, mmol/L	5.32 ± 1.11	5.16 ± 0.87	5.44 ± 1.24
UpH	6.12 ± 0.65	6.17 ± 0.66	6.08 ± 0.64

Abbreviations: BMI, body mass index; SBP, systolic blood pressure; DBP, diastolic blood pressure; ALT, alanine aminotransferase; AST, aspartate aminotransferase; TP, total protein; Alb, albumin; Glo, globulin; GGT, γ-glutamyl transpeptidase; SCr, serum creatinine; eGFR, estimated glomerular filtration rate; TBIL, total bilirubin; IBIL, indirect bilirubin; DBIL, direct bilirubin; TC, total cholesterol; HDL, high-density lipoprotein cholesterol; LDL, low-density lipoprotein cholesterol; TG, triglycerides; UA, uric acid; Glu, fasting glucose; UpH, Urine pH; OR, odds ratio; CI, confidence interval.

All *p* value < .001.

^a^Structures reported by ultrasonography examination were regarded as kidney stones regardless of the size.

^b^Calculated as weight in kilograms divided by height in metres squared.

^c^Calculated using the CKD-EPI equation (Details can be found in the Methods section).

Table 2 demonstrates the patient factors and the odds ratio of presenting urolithiasis stratified by sex. Through multivariate-adjusted logistic regression, partial factors may influence the occurrence of kidney stones differently between the two genders. The results indicate that presenting higher levels of LDL, TG, UA, and Glu were shown to be risk factors for males. Higher levels of eGFR might be protective factors for males. Age, HBP, FL, and higher UpH were associated to a higher risk of urolithiasis in both genders.

**Table 2.** Patient factors and the odds ratio of presenting urolithiasis stratified by sex.

	OR (95% CI)			
	Univariate		Multivariate-adjusted^a^	
Variables	Female	Male	Female	Male
Age	1.020 (1.018–1.023)***	1.016 (1.014–1.018)***	1.020 (1.015–1.025)***	1.010 (1.008–1.013)***
Obesity	1.171 (1.080–1.269)***	1.306 (1.244–1.371)***	0.916 (0.833–1.005)	1.056 (0.999–1.117)
HBP	1.715 (1.510–1.942)***	1.621 (1.511–1.738)***	1.160 (1.002–1.340)*	1.184 (1.094–1.282)***
DM	1.535 (1.190–1.951)***	1.376 (1.212–1.556)***	1.004 (0.750–1.328)	0.963 (0.834–1.110)
CHD	1.627 (0.976–2.557)*	1.388 (1.058–1.794)*	0.917 (0.543–1.467)	0.944 (0.715–1.231)
FL	1.487 (1.351–1.635)***	1.319 (1.258–1.384)***	1.301 (1.159–1.457)***	1.128 (1.068–1.192)***
eGFR	0.989 (0.987–0.991)***	0.987 (0.986–0.989)***	1.002 (0.998–1.005)	0.995 (0.993–0.997)***
HDL	0.892 (0.788–1.008)	0.776 (0.705–0.854)***	1.026 (0.896–1.175)	1.008 (0.906–1.121)
LDL	1.161 (1.106–1.219)***	1.093 (1.060–1.128)***	1.048 (0.994–1.103)	1.060 (1.026–1.094)***
TG	1.106 (1.067–1.145)***	1.060 (1.045–1.074)***	1.030 (0.981–1.076)	1.021 (1.004–1.038)*
UA	1.023 (0.988–1.058)	1.127 (1.109–1.145)***	0.974 (0.937–1.012)	1.107 (1.087–1.127)***
Glu	1.079 (1.042–1.117)***	1.077 (1.060–1.095)***	0.977 (0.931–1.023)	1.042 (1.021–1.063)***
UpH	1.048 (0.992–1.108)	0.940 (0.906–0.975)**	1.073 (1.014–1.135)*	1.059 (1.020–1.101)**

Abbreviations: HBP, hypertension; DM, diabetes mellitus; CHD, Coronary heart disease; FL, fatty liver; eGFR, estimated glomerular filtration rate; TC, total cholesterol; HDL, high-density lipoprotein cholesterol; LDL, low-density lipoprotein cholesterol; TG, triglycerides; UA, uric acid; Glu, fasting glucose; UpH, Urine pH; OR, odds ratio; CI, confidence interval.

^a^Adjusted as model 2 (see Methods-Statistical Analyses section for descriptions of model 2).

*p < .05; **p < .01; ***p < .001.

**Figure 2.**
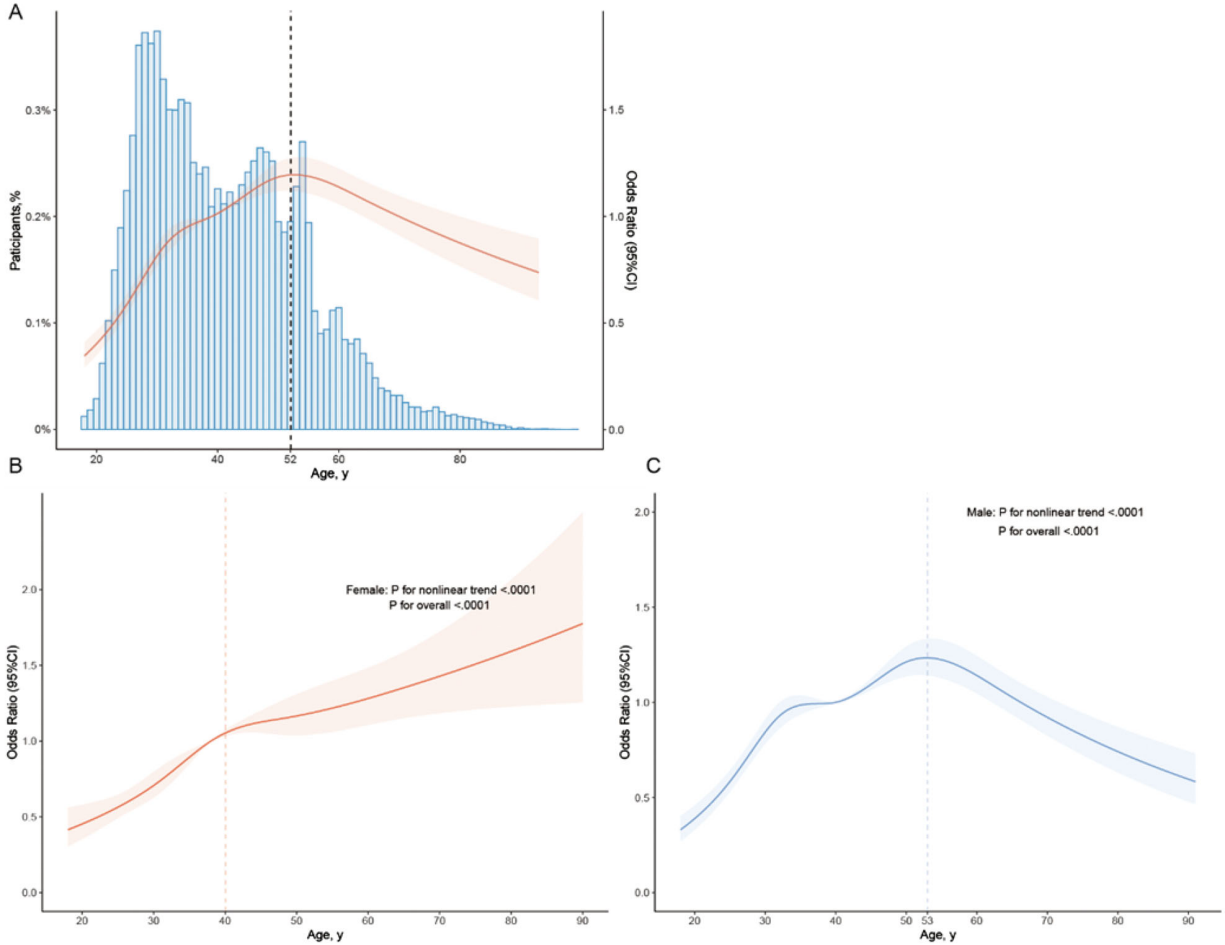
Association between age and urolithiasis. Adjusted as model 2 (see Methods-Statistical Analyses section for a description of model 2), restricted cubic splines were applied (details can be found in the Methods-Statistical Analyses section). (A) Odds ratio (OR) with 95% confidence interval (95%CI) was indicated by the red line and shade; blue histogram illustrated the distribution of the whole participants. The dashed line demonstrated the inflection point where the OR for urolithiasis reversed the trend. (B) Stratified by sex, the red and blue line and shade presented OR and 95%CI of urolithiasis in females and in males, respectively. The risk for urolithiasis in females continuously rose as ageing, while for males the risk indicated a trend to ascend until the age of around 53 and then descend.

Therefore, we next characterised the influence of age on urolithiasis. Applying an extended model approach, the OR for urolithiasis stratified by sex presents distinct trends between different genders. The ORs for females manifest a trend to increase continuously as the age grows. Nevertheless, The ORs for females show a trend to increase first and then decrease. The change in the prevalence of urolithiasis in different age groups is similar to that of OR, but the prevalence of urolithiasis in males (≥75 years old) is still higher than in females (≥75 years old) although the risk decreases at the last (Table 3). Consequently, we analysed the distribution and the changing trend of OR for urolithiasis along with the age growing fitting the restricted cubic spline, coming out that for the whole population the risk for urolithiasis rises with the age growing before the age of 52 (Figure 2A). Separate analyses of the two genders corresponded with the results mentioned above: the risk for urolithiasis in females continuously rises as ageing (Figure 2B), while for males the risk presents a trend to ascend until the age of around 53 and then descend (Figure 2C).

**Table 3.** The odds ratio for urolithiasis stratified by sex and age.

Stratified age (y)	No. of participants presenting urolithiasis/total participants (%)	Unadjusted	Model 1-adjusted^a^	Model 2-adjusted^a^
Female				
18–29	505/10700 (4.72)	Ref.	Ref.	Ref.
30–44	1149/16413 (7.00)	1.520 (1.366–1.693)***	1.504 (1.351–1.677)***	1.463 (1.307–1.639)***
45–59	1001/11706 (8.55)	1.888 (1.691–2.109)***	1.796 (1.603–2.014)***	1.690 (1.469–1.946)***
60–74	348/3333 (10.44)	2.354 (2.040–2.713)***	2.103 (1.800–2.455)***	1.959 (1.605–2.389)***
≥75	73/610 (11.97)	2.744 (2.101–3.538)***	2.403 (1.803–3.166)***	2.244 (1.606–3.110)***
Male				
18–29	966/10515 (9.19)	Ref.	Ref.	Ref.
30–44	3211/22580 (14.22)	1.639 (1.519–1.769)***	1.551 (1.437–1.676)***	1.501 (1.388–1.624)***
45–59	3093/17241 (17.94)	2.161 (2.002–2.334)***	1.936 (1.790–2.097)***	1.801 (1.650–1.967)***
60–74	743/4232 (17.56)	2.105 (1.898–2.334)***	1.830 (1.642–2.039)***	1.631 (1.443–1.843)***
≥75	122/902 (13.53)	1.546 (1.258–1.885)***	1.296 (1.047–1.592)*	1.031 (0.820–1.288)

^a^Adjusted as model 1,2 (see Methods-Statistical Analyses section for descriptions of model 2), restricted cubic splines were applied (details can be found in the Methods-Statistical Analyses section).

*p < .05; **p < .01; ***p < .001.

**Figure 3.**
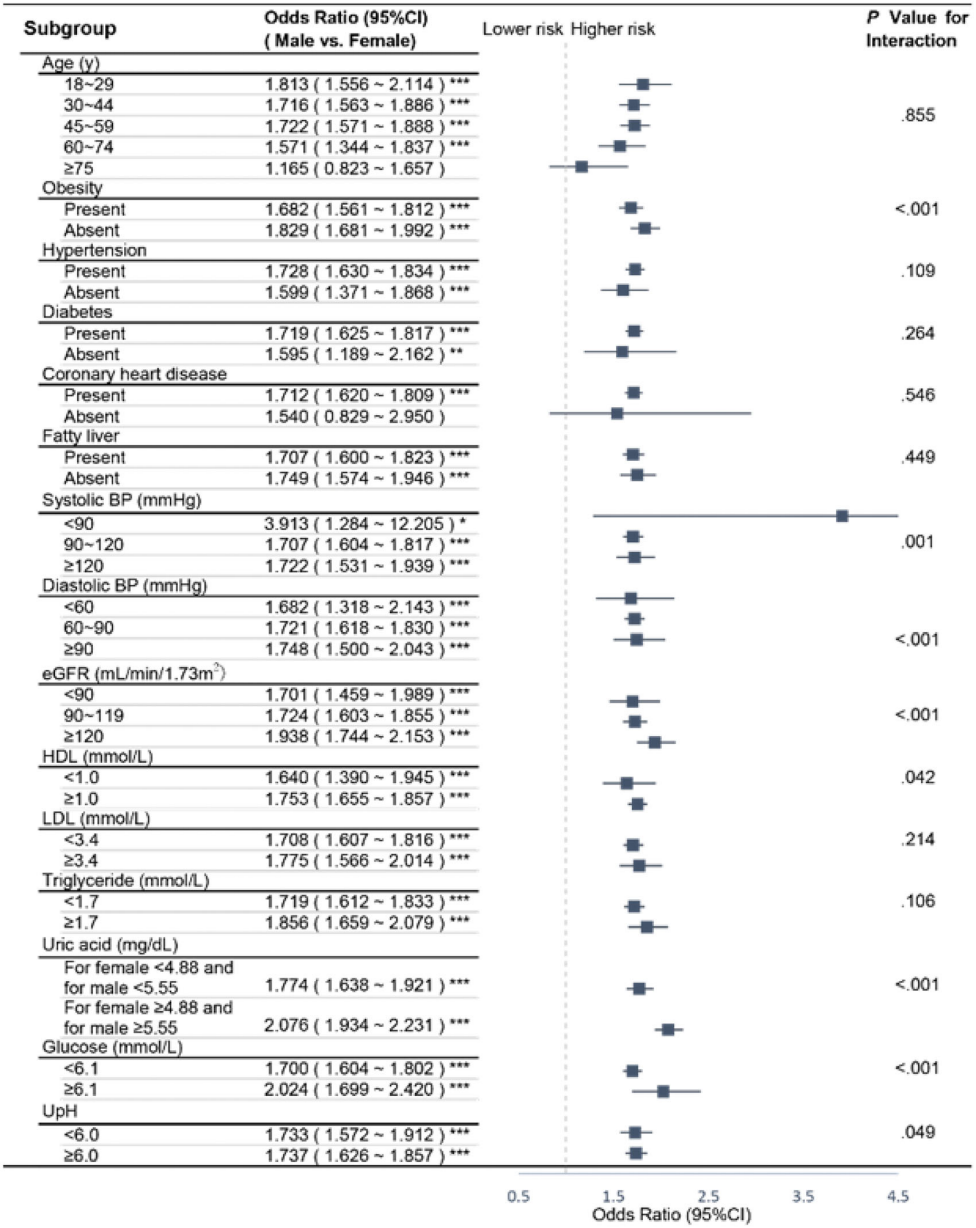
Subgroup analyses on the OR of sex (male vs. female). Abbreviations: CI, confidence interval; BP, blood pressure; eGFR, estimated glomerular filtration rate; HDL, high-density lipoprotein; LDL, low-density lipoprotein; UpH, Urine pH. Adjusted as model 2 (see Methods-Statistical Analyses section for descriptions of model 2). *P* for interaction was calculated applying the Wald test. Except for those who are older than 75 years old or absent of CHD, the risk of males developing kidney stones is significantly higher than that of females. *p < .05; **p < .01; ***p < .001.

We further conducted subgroup analyses by calculating the OR (95%CI) for urolithiasis in males in reference to females, finding that except for those who are older than 75 years old or absent of CHD, the risk of males developing kidney stones is significantly higher than that of females (Figure 3). With the assistance of propensity score matching, we could to the greatest extent overcome the interference of bias and confounding factors. After proper sex matching (sTable 2), we repeated the investigations on the 34876 matched participants in the OR for presenting urolithiasis of diverse patient factors and stratified by sex (sTable 3). Without matching age (sTable 4), we then illustrated the association between the OR of urolithiasis and age, again between the two genders coming out with similar trends (sFigure). Furthermore, we excluded the samples whose stones were reported to be <3mm and repeated the analysis (sTable 5).

## Discussion

In this cross-sectional study, we concentrated on the sex disparities in urolithiasis. It was observed that patients’ factors might cast an influence on the development of kidney stone disease distinctly between the two genders. A risk factor for one gender might have no effect on urolithiasis for the other gender. Although age seemed to be a risk factor for both genders, further analyses demonstrated that the risk to develop kidney stones in females continuously ascended, while the risk in males presented a trend to ascend and then descend as age grew. Despite that, the risk of males for urolithiasis decreased, at last, the prevalence of urolithiasis was still higher in males than in females.

Presenting higher levels of LDL, TG, UA, and Glu were shown to be risk factors for males. Higher levels of eGFR might be protective factors for males. Former researchers have demonstrated how diverse factors contributed to urolithiasis distinctly. Seolhye et al. reported that increased serum uric acid levels, higher glycemic values, and Homeostasis Model Assessment of Insulin Resistance (HOMA-IR) were associated with increased risk for the development of urolithiasis in a dose-response manner in males but not in females, and attributed such sex differences to the protective effects of the female hormone [12,13]. It was also indicated that males were more vulnerable to urolithiasis when exposed to ambient temperatures, which might be explicated by the sexually dimorphic renal physiology [14]. Using a classic twin study, it was stated urolithiasis in females had a heritable component less than that in males, and environmental risk factors might play a greater role in females [15]. Gene exploration described the association of *HIPK2* gene polymorphisms with urolithiasis in males but not in females, and *HIPK2* showed a relation to systolic blood pressure, creatinine, and uric acid levels [16]. Studies on distinct contributions of these factors may provide evidence for sex-specific clinical strategy.

Furthermore, we found as age grew, the risk for urolithiasis presented different trends in the two genders. According to Hui et al., based on data from the Global Burden of Disease Study 2019, for either sex, the urolithiasis incidence in China increased and then decreased with ageing [17]. Qiang et al. reported that the prevalence rates of both genders increased first and then decreased as ageing in southern China, while it rase with age growing constantly in northern China [18]. Nevertheless, as age grew, the difference in risk between the two genders was narrowing in both types of research, which seemed to correspond to our research. Our results also demonstrated that the risk of males developing kidney stones was around 1.68 times the risk of females. However, for those who were older than 75 years old, the disparities between the sexes were no longer significant.

Sex steroid differences that result in distinct conditions between males and females might be the physiological basis underlying gender differences [19]. A possible explanation for the inflection in the association between OR of urolithiasis and age in males might be the alteration of the androgen signals. Ageing is related to a progressive decrease in testosterone levels, meanwhile, the androgen signal has been widely discussed to be a risk factor for urolithiasis. The expression of androgen receptors in the kidney of patients with renal calculi was significantly up-regulated [20]. Exogenous testosterone could elevate the risk of stone events, while androgen deprivation therapy and finasteride reduce the risk of kidney stones [21]. From the perspective of mechanism, testosterone could increase α-enolase expression on the surface of renal tubular cells leading to adhesion of crystals to cells, and loss of the androgen receptor could suppress intrarenal crystals deposition *via* altering macrophage recruitment with alteration of the *miR-185-5p/CSF-1* signals [22,23]. The reason underlying the change of risk in males might be the gradual weakening of androgen signal levels after a certain age. However, the contribution of oestrogen remained controversial. Postmenopausal status was reported to be associated with a higher risk of incident kidney stones [24]. Mechanically oestrogen was manifested to prevent urolithiasis *via* inhibiting the oxalate biosynthesis and renal injury [25], or *via* enhancing cell proliferation and tissue healing [26]. Nevertheless, other researchers have indicated that menopause or oestrogen might have little association with the risk of urolithiasis [27]. Our results were not capable of corroborating the protective function of oestrogen either, as no inflection to an accelerated risk accumulation for urolithiasis in females was observed. Since the imaging techniques were able to discover asymptomatic stones, the risk to be diagnosed with urolithiasis ascended as ageing in females might be a result of hazard accumulation over time [11]. To elaborate on the contribution of sex hormones especially oestrogen to the development of kidney calculi required further evidence. Our findings may add to the burgeoning understanding that sex should be considered a biologically relevant variable and an important determinant of health outcomes. We expect that more studies can cast light on the sex disparities in urolithiasis to preferably guide precise prevention and treatment.

In addition, HBP, FL, and UpH presented a positive correlation with a higher risk for urolithiasis in both genders. Hypertension was reported to be associated with an increased risk of urolithiasis from epidemiological and genetic perspectives [28]. Federico et al. also confirmed a relationship between urolithiasis and fatty liver disease [29]. Dyslipidemia was stated to be risk factor for urolithiasis and could serve as the bridge between urolithiasis and these systemic diseases [30]. However, blood lipids have been adjusted in our models and other crosstalk existed between these diseases and urolithiasis. Inflammatory network and immune status could also play a vital role in such association [31]. Fatma et al. demonstrated that nucleation kinetics of calcium oxalate monohydrate was dramatically slower at pH 6.0 compared to pH 3.6 and pH 8.6 [32]. In our participants, the mean UpH was around 6.0 and a higher pH could be a risk factor for urolithiasis. In the meanwhile, stone obstruction, urological procedures, and urine alkalisation for stone prevention could increase the UpH, which could from another perspective explain the positive association between urolithiasis and higher UpH [33].

This study still has several limitations. First, the study was designed as a cross-sectional survey, thus could explain little causality. Nevertheless, we concentrated on age this non-modifiable factor and established a dose-response relationship applying restricted cubic splines. Second, urolithiasis was diagnosed by ultrasonography rather than computed tomography. However, as a radiation-free and low-cost imaging method, ultrasonography was strongly recommended for screening in a large population and we also performed sensitivity analyses to minimise the impact of the low accuracy of ultrasonography [34]. Third, we lacked the analysis of the composition of stones. To compensate for that, we adjusted UpH to reflect the diverse stone types and urine chemistry [35]. Fourth, no information about the prior history and treatment of stone disease was obtained. Fifth, no dietary information and the history of other co-morbidities such as gout and bowel disease were obtained. Sixth, our analyses depended on single-centre physical examination information. Hence, it was arduous to eliminate the bias and further multicenter prospective studies were warranted.

## Conclusions

Patients’ factors might cast an influence on the development of kidney stone disease distinctly between the two genders. Presenting DM, DBP, LDL, UA, and Glu may be risk factors for males but not for females. SBP and eGFR may serve as a protective factor for males but not for females. As age grew, the risk to develop kidney stones in females continuously ascended, while the risk in males presented a trend to ascend and then descend, which was presumably related to the weakening of the androgen signal.

## Supplementary Material

Supplemental Material

## Data Availability

Our data is not publicly available considering that individual privacy could be compromised, but are available from the corresponding author on reasonable request.

## Abbreviations

HBP: hypertension
DM: diabetes mellitus
CHD: Coronary heart disease
FL: fatty liver
BMI: body mass index
SBP: systolic blood pressure
DBP: diastolic blood pressure
ALT: alanine aminotransferase
AST: aspartate aminotransferase
TP: total protein
Alb: albumin
Glo: globulin
GGT: γ-glutamyl transpeptidase
SCr: serum creatinine
eGFR: estimated glomerular filtration rate
TBIL: total bilirubin
IBIL: indirect bilirubin
DBIL: direct bilirubin
TC: total cholesterol
HDL: high-density lipoprotein cholesterol
LDL: low-density lipoprotein cholesterol
TG: triglycerides
UA: uric acid
Glu: fasting glucose
UpH: Urine pH
OR: odds ratio
CI: confidence interval
