# Supplementary material for: Sex disparities and the risk of urolithiasis: a large cross-sectional study
Source: Ann Med. 2022 Jun 8;54(1):1627–35. doi: 10.1080/07853890.2022.2085882 (PMC9196832; doi:10.1080/07853890.2022.2085882)
Supplement: Supplemental Material [file IANN_A_2085882_SM2959.zip › Supplemental files/Supplementary materials_re1.docx]

sTable 1. Comparation of Basic Characteristics before and after Multiple Imputation.

| **Variables** | | Before MI | After MI | *P* value |
| --- | --- | --- | --- | --- |
| Presenting Characteristics | |  |  |  |
|  | BMI, kg/m^2^ | 23.56 ± 3.39 | 23.55 ± 3.38 | 0.83 |
|  | SBP, mmHg | 123.87 ± 17.97 | 123.83 ± 17.94 | 0.64 |
|  | DBP, mmHg | 75.82 ± 12.04 | 75.79 ± 12.03 | 0.59 |
| Laboratory indices | |  |  |  |
|  | ALT, U/L | 23.32 ± 22.22 | 23.32 ± 22.2 | 0.91 |
|  | AST, U/L | 21.96 ± 12.53 | 21.95 ± 12.46 | 0.83 |
|  | TP, g/L | 76.02 ± 3.92 | 76.02 ± 3.96 | 0.85 |
|  | Alb, g/L | 46.12 ± 2.58 | 46.11 ± 2.6 | 0.83 |
|  | Glo, g/L | 29.9 ± 3.54 | 29.91 ± 3.57 | 0.67 |
|  | GGT, U/L | 31.19 ± 35.14 | 31.05 ± 34.72 | 0.43 |
|  | Scr, μmol/L | 73.79 ± 18.52 | 73.86 ± 18.48 | 0.27 |
|  | eGFR, mL/min/1.73m^2^ | 111.97 ± 17.25 | 112.02 ± 17.23 | 0.60 |
|  | TBIL, μmol/L | 13.64 ± 5.46 | 13.63 ± 5.47 | 0.78 |
|  | IBIL, μmol/L | 9.96 ± 4.04 | 9.96 ± 4.11 | 0.91 |
|  | DBIL, μmol/L | 3.67 ± 1.71 | 3.67 ± 1.7 | 0.54 |
|  | TC, mmol/L | 4.53 ± 0.87 | 4.53 ± 0.87 | 0.79 |
|  | HDL, mmol/L | 1.28 ± 0.31 | 1.28 ± 0.31 | 0.93 |
|  | LDL, mmol/L | 2.73 ± 0.75 | 2.73 ± 0.75 | 0.69 |
|  | TG, mmol/L | 1.47 ± 1.28 | 1.47 ± 1.28 | 0.90 |
|  | UA, mg/dL | 341.75 ± 95.58 | 342.12 ± 95.54 | 0.34 |
|  | Glu, mmol/L | 5.33 ± 1.12 | 5.32 ± 1.11 | 0.096 |
|  | UpH | 6.12 ± 0.65 | 6.12 ± 0.65 | 0.67 |

**Abbreviations**: MI, multiple imputation; BMI, body mass index; SBP, systolic blood pressure; DBP, diastolic blood pressure; ALT, alanine aminotransferase; AST, aspartate aminotransferase; TP, total protein; Alb, albumin; Glo, globulin; GGT, γ-glutamyl transpeptidase; SCr, serum creatinine; eGFR, estimated glomerular filtration rate; TBIL, total bilirubin; IBIL, indirect bilirubin; DBIL, direct bilirubin; TC, total cholesterol; HDL, high-density lipoprotein cholesterol; LDL, low-density lipoprotein cholesterol; TG, triglycerides; UA, uric acid; Glu, fasting glucose; UpH, Urine pH;

No. of participants have missing data for the variables: BMI, n=4839; blood pressure, n=3615; ALT, n=1498; AST, n=2646; TP, Alb, n=9944; Glo, n=9969; GGT, n=8338; SCr, n=1478; TBIL, n=7053; IBIL, n=7998; DBIL,7899; TC, TG, n=3344; HDL, LDL n=3901; UA, n=3349; Glu, n=3261; UpH, n=3683.

P Values were calculated using one-way analysis of variance.

sTable 2 Comparation of Characteristics after Propensity Score Matching.

| Variables | | All Participants | Female | Male | P Value |
| --- | --- | --- | --- | --- | --- |
|  |  | (n= 35456) | (n= 17728) | (n= 17728) |  |
|  | Urolithiasis present (%) | 3654(10.3) | 1301(7.3) | 2353(13.3) | <0.001 |
|  | Age, y | 42.77 ± 13.86 | 42.74 ± 14.29 | 42.79 ± 13.41 | 0.733 |
| Comorbidity | | | | | |
|  | Obesity present (%) | 14361(40.5) | 7247(40.9) | 7114(40.1) | 0.153 |
|  | Hypertension present (%) | 3366(9.5) | 1675(9.4) | 1691(9.5) | 0.786 |
|  | Diabetes present (%) | 1035(2.9) | 504(2.8) | 531(3.0) | 0.412 |
|  | Coronary heart disease present (%) | 241(0.7) | 119(0.7) | 122(0.7) | 0.897 |
|  | Fatty liver present (%) | 8240(23.2) | 4116(23.2) | 4124(23.3) | 0.930 |
| Laboratory indices | | | | |  |
|  | eGFR, mL/min/1.73m2 | 112.04 ± 16.83 | 112.21 ± 18.65 | 111.87 ± 14.80 | 0.057 |
|  | HDL, mmol/L | 1.29 ± 0.27 | 1.29 ± 0.25 | 1.29 ± 0.28 | 0.650 |
|  | LDL, mmol/L | 2.73 ± 0.75 | 2.74 ± 0.76 | 2.73 ± 0.73 | 0.516 |
|  | TG, mmol/L | 1.38 ± 1.19 | 1.38 ± 1.05 | 1.38 ± 1.31 | 0.910 |
|  | UA, mg/dL | 5.44 ± 1.01 | 5.42 ± 1.02 | 5.46 ± 1.01 | <0.001 |
|  | Glu, mmol/L | 5.34 ± 1.10 | 5.34 ± 1.15 | 5.34 ± 1.05 | 0.942 |
|  | UpH | 6.13 ± 0.65 | 6.13 ± 0.66 | 6.12 ± 0.65 | 0.402 |

Abbreviation: eGFR, estimated glomerular filtration rate; HDL, high-density lipoprotein cholesterol; LDL, low-density lipoprotein cholesterol; TG, triglycerides; UA, uric acid; Glu, fasting glucose; UpH, Urine pH.

P Values were calculated using one-way analysis of variance for continuous variables, and chi-square test for categorical variables.

sTable 3. OR of Urolithiasis adjusted for patient factors after Propensity Score Matching

| **Variables** | | **OR (95%CI)** | |
| --- | --- | --- | --- |
|  |  | Female | Male |
|  | age | 1.017 (1.011 ~ 1.024) *** | 1.011 (1.006 ~ 1.016) *** |
|  | Obesity | 0.894 (0.786 ~ 1.017) | 1.059 (0.958 ~ 1.171) |
|  | HBP | 1.366 (1.128 ~ 1.647) ** | 1.204 (1.038 ~ 1.393) * |
|  | DM | 0.976 (0.689 ~ 1.360) | 0.930 (0.708 ~ 1.210) |
|  | CHD | 0.916 (0.491 ~ 1.589) | 1.007 (0.605 ~ 1.600) |
|  | FL | 1.162 (1.002 ~ 1.346) * | 1.176 (1.050 ~ 1.316) ** |
|  | eGFR | 1.002 (0.997 ~ 1.007) | 0.999 (0.995 ~ 1.003) |
|  | HDL | 0.925 (0.720 ~ 1.184) | 0.981 (0.828 ~ 1.161) |
|  | LDL | 1.047 (0.970 ~ 1.129) | 1.043 (0.982 ~ 1.108) |
|  | TG | 1.003 (0.942 ~ 1.061) | 1.000 (0.964 ~ 1.034) |
|  | UA | 1.041 (0.982 ~ 1.103) | 1.089 (1.041 ~ 1.139) *** |
|  | Glu | 0.990 (0.935 ~ 1.044) | 1.039 (0.992 ~ 1.086) |
|  | UpH | 1.018 (0.933 ~ 1.111) | 1.111 (1.037 ~ 1.189) ** |

**Abbreviations**: HBP, hypertension; DM, diabetes mellitus; CHD, Coronary heart disease; FL, fatty liver; eGFR, estimated glomerular filtration rate; TC, total cholesterol; HDL, high-density lipoprotein cholesterol; LDL, low-density lipoprotein cholesterol; TG, triglycerides; UA, uric acid; Glu, fasting glucose; UpH, Urine pH; OR, odds ratio; CI, confidence interval.

Adjusted as model 2 (see Methods-Statistical Analyses section for descriptions of model 2).

* *p*<0.05; ** *p*<0.01; *** *p*<0.001.

sTable 4. Comparation of Characteristics after Propensity Score Matching. (without matching age).

| **Variables** | | All Participants | Female | Male | *P* Value |
| --- | --- | --- | --- | --- | --- |
|  |  | (n=35566) | (n=17783) | (n=17783) |  |
|  | Urolithiasis present (%) | 3653(10.3) | 1340(7.5) | 2313(13.0) | <0.001 |
|  | Age, y | 42.62 ± 13.74 | 43.41 ± 14.31 | 41.82 ± 13.09 | <0.001 |
| Comorbidity | | | | | |
|  | Obesity present (%) | 14519(40.8) | 7329(41.2) | 7190(40.4) | 0.137 |
|  | Hypertension present (%) | 3358(9.4) | 1700(9.6) | 1658(9.3) | 0.457 |
|  | Diabetes present (%) | 1026(2.9) | 508(2.9) | 518(2.9) | 0.776 |
|  | Coronary heart disease present (%) | 244(0.7) | 117(0.7) | 127(0.7) | 0.563 |
|  | Fatty liver present (%) | 8393(23.6) | 4165(23.4) | 4228(23.8) | 0.439 |
| Laboratory indices | | | | |  |
|  | eGFR, mL/min/1.73m^2^ | 112.02 ± 16.77 | 112.16 ± 18.55 | 111.87 ± 14.79 | 0.108 |
|  | HDL, mmol/L | 1.29 ± 0.27 | 1.29 ± 0.25 | 1.29 ± 0.28 | 0.700 |
|  | LDL, mmol/L | 2.74 ± 0.75 | 2.74 ± 0.77 | 2.74 ± 0.73 | 0.595 |
|  | TG, mmol/L | 1.38 ± 1.17 | 1.38 ± 1.01 | 1.38 ± 1.31 | 0.742 |
|  | UA, mg/dL | 5.45 ± 1.01 | 5.42 ± 1.02 | 5.47 ± 1.01 | <0.001 |
|  | Glu, mmol/L | 5.34 ± 1.10 | 5.35 ± 1.13 | 5.34 ± 1.07 | 0.696 |
|  | UpH | 6.13 ± 0.65 | 6.13 ± 0.66 | 6.12 ± 0.65 | 0.400 |

Abbreviation: eGFR, estimated glomerular filtration rate; HDL, high-density lipoprotein cholesterol; LDL, low-density lipoprotein cholesterol; TG, triglycerides; UA, uric acid; Glu, fasting glucose; UpH, Urine pH.

P Values were calculated using one-way analysis of variance for continuous variables, and chi-square test for categorical variables.

sTable 5. OR of Urolithiasis adjusted for patient factors after excluding samples whose stones were ≤3mm.

| **Variables** | | **OR (95%CI)** | |
| --- | --- | --- | --- |
|  |  | Female | Male |
|  | age | 1.020 (1.016 ~ 1.025) *** | 1.012 (1.009 ~ 1.014) *** |
|  | Obesity | 0.913 (0.830 ~ 1.004) | 1.061 (1.003 ~ 1.122) * |
|  | HBP | 1.158 (0.998 ~ 1.340) | 1.181 (1.090 ~ 1.279) *** |
|  | DM | 0.980 (0.729 ~ 1.300) | 0.970 (0.839 ~ 1.118) |
|  | CHD | 0.936 (0.554 ~ 1.497) | 0.929 (0.701 ~ 1.215) |
|  | FL | 1.280 (1.139 ~ 1.437) *** | 1.113 (1.052 ~ 1.177) *** |
|  | eGFR | 1.002 (0.998 ~ 1.006) | 0.995 (0.993 ~ 0.997) *** |
|  | HDL | 1.046 (0.912 ~ 1.200) | 0.999 (0.896 ~ 1.112) |
|  | LDL | 1.050 (0.996 ~ 1.107) | 1.064 (1.030 ~ 1.100) *** |
|  | TG | 1.035 (0.986 ~ 1.081) | 1.026 (1.009 ~ 1.042) ** |
|  | UA | 0.976 (0.938 ~ 1.015) | 1.107 (1.087 ~ 1.128) *** |
|  | Glu | 0.984 (0.937 ~ 1.030) | 1.041 (1.020 ~ 1.063) *** |
|  | UpH | 1.075 (1.015 ~ 1.137) * | 1.061 (1.021 ~ 1.103) ** |

**Abbreviations**: HBP, hypertension; DM, diabetes mellitus; CHD, Coronary heart disease; FL, fatty liver; eGFR, estimated glomerular filtration rate; TC, total cholesterol; HDL, high-density lipoprotein cholesterol; LDL, low-density lipoprotein cholesterol; TG, triglycerides; UA, uric acid; Glu, fasting glucose; UpH, Urine pH; OR, odds ratio; CI, confidence interval.

Adjusted as model 2 (see Methods-Statistical Analyses section for descriptions of model 2).

* *p*<0.05; ** *p*<0.01; *** *p*<0.001.
