# Supplementary figures and images for: Sex disparities and the risk of urolithiasis: a large cross-sectional study
Source: Ann Med. 2022 Jun 8;54(1):1627–35. doi: 10.1080/07853890.2022.2085882 (PMC9196832; doi:10.1080/07853890.2022.2085882)

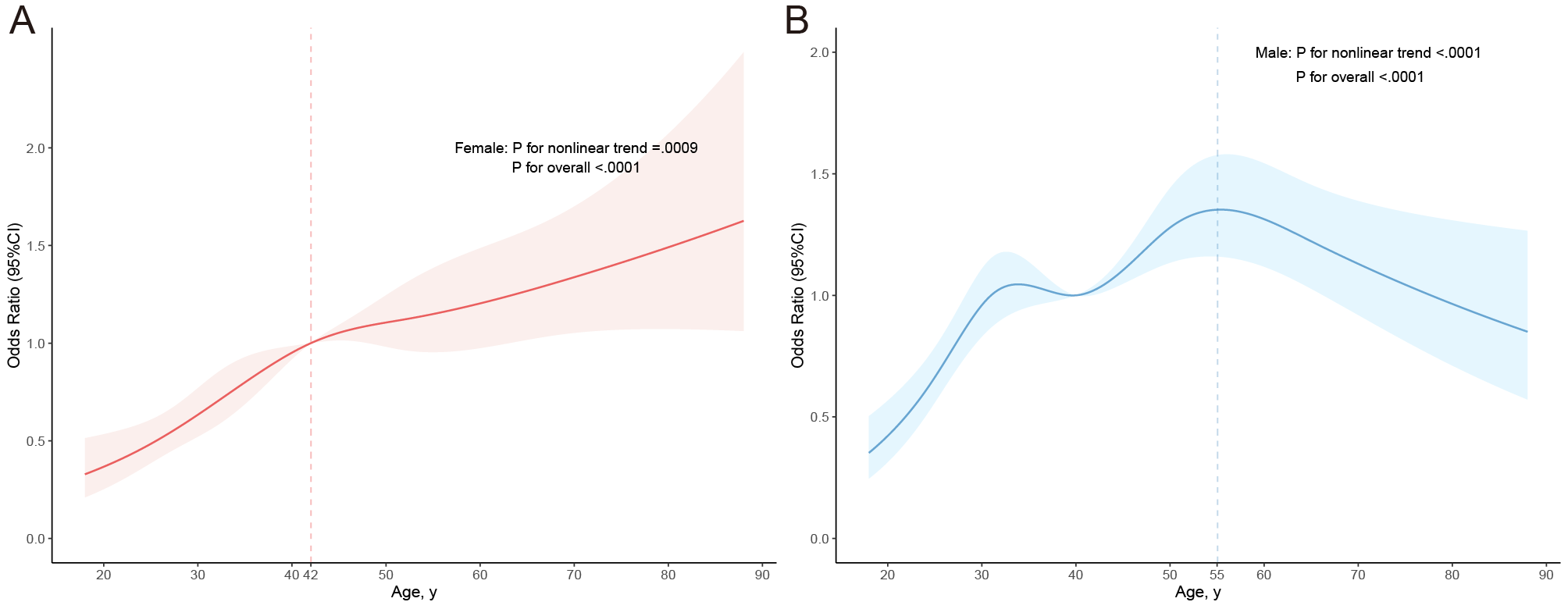

Supplement: Supplemental Material [file IANN_A_2085882_SM2959.zip › Supplemental files/sFigure 1.png]
